# Refining the diagnostic evaluation of idiopathic normal pressure hydrocephalus with Alzheimer's and α-synuclein biomarkers

**DOI:** 10.3389/fneur.2026.1736687

**Published:** 2026-03-17

**Authors:** Vivienne A. Wluka, Tyrone Despenza, Andy J. Liu, Alexa N. Bramall

**Affiliations:** 1 Department of Neurosurgery, Duke University Hospital, Durham, NC, United States; 2 Department of Neurology, Duke University Hospital, Durham, NC, United States

**Keywords:** Alzheimer's disease, biomarkers, cerebrospinal fluid, idiopathic normal pressure hydrocephalus, α-synuclein

## Abstract

Idiopathic normal pressure hydrocephalus (iNPH), characterized by ventriculomegaly and Hakim's triad of gait disturbance, cognitive impairment, and urinary dysfunction, is common in older adults. iNPH also remains one of the few potentially reversible neurological conditions, typically treated with cerebrospinal fluid (CSF) shunting. Although many patients improve, shunt responsiveness varies widely (≈60%−90%), and a subset of patients show only transient benefit. A major contributor to this variability is the high prevalence of comorbid neurodegenerative disease with symptom overlap, particularly Alzheimer's disease (AD) and α-synucleinopathies such as Parkinson's disease (PD) and dementia with Lewy bodies (DLB). The impact of these concomitant conditions on shunt outcomes, however, remains uncertain. We describe the incorporation of AD CSF biomarkers and α-synuclein skin biopsy into the iNPH evaluation to identify coexisting pathology. When integrated into routine workflow, approximately 32% of patients demonstrated AD-consistent CSF profiles and 31% had biopsy-confirmed α-synuclein pathology. We propose adopting concomitant neurological testing, especially in patients with atypical features to help inform patient selection and guide expectations. As the awareness and potential prevalence of iNPH rises and shunt procedures carry meaningful complication risks, delineating how comorbid disease modifies outcomes will be essential to improving the long-term success of shunting in iNPH.

## Introduction

Idiopathic normal pressure hydrocephalus (iNPH), defined by the triad of gait disturbance, cognitive impairment, and urinary incontinence with ventriculomegaly on neuroimaging, is relatively common in older adults. Population estimates suggest a prevalence of approximately 3.7% among individuals ≥ 65 years of age (1), rising to 7.7% among those aged 86 years and older (2). In nursing homes, the prevalence has been estimated at 14% (3). Although recognition of iNPH as a true and reversible clinical entity has gained support over time, now confirmed by the placebo-controlled efficacy of iNPH shunting trial (4, 5), it is estimated that up to 80% of cases remain undiagnosed and untreated (6). Nevertheless, treatment for iNPH significantly improves quality of life (7, 8) and may also prolong life expectancy, potentially by lowering fall risk (9). Identifying patients with iNPH and accurately selecting patients who may respond positively to treatment is therefore a key clinical priority. Currently, two clinical guidelines exist for iNPH, with slightly different diagnostic criteria (10, 11).

Even with advanced imaging, gait assessment, and invasive provocative tests, the probability of shunt responsiveness, broadly defined as an objective or subjective improvement in the clinical symptoms of iNPH following shunt surgery, ranges from 60 to 90% (12, 13). However, there is a substantial risk of shunt-related complications, including device failure, infection, and subdural hematoma, the latter complication occurring in around 10% of patients (14). Moreover, among patients who initially show benefits from shunting, approximately 20% may experience secondary deterioration within 3 years of placement (15). One explanation for this deterioration is unrecognized co-existing neurodegenerative disorders (16), such as Alzheimer's disease (AD) or α-synucleinopathies [most commonly Parkinson's disease (PD) and dementia with Lew bodies (DLB)], which may limit the extent and duration of shunting benefits. It is therefore prudent to assess for these conditions alongside the evaluation for iNPH. The aim of this paper is to present our results applying an α-synuclein skin biopsy and AD cerebrospinal fluid (CSF) biomarkers to patients with suspected iNPH and outline a suggested pathway for evaluation. By identifying comorbid neurological disorders, we can help stratify the iNPH patient population to understand how these conditions influence short- and long-term shunt responsiveness and, in turn, inform patient expectations and outcomes in iNPH.

## A heterogeneous population with high frequency of comorbid neurological disorders

One of the central challenges in diagnosing iNPH is that its presenting features overlap substantially with conditions common in older adults. For example, one third of individuals 65 years of age or older have dementia or mild cognitive impairment (17). By the age of 75, 30%−50% of people have urinary incontinence (18), and 35% of individuals have gait abnormalities after the age of 70 (19). As a result, iNPH is often missed as a possible diagnosis since many of the presenting symptoms are attributed to normal aging. Conversely, some patients are referred after ventriculomegaly is detected incidentally, often following hospitalization for falls or other medical concerns. Yet ventriculomegaly is relatively common in older adults: in a population-based cohort of Swedish 70-year-olds, 11% had an Evans' index >0.3 without other radiographic criteria for iNPH (20). The presence of one or two triad features often triggers a full evaluation, which varies considerably by center, and may lead to surgical intervention. Furthermore, without prior imaging, the chronicity of ventriculomegaly cannot be determined, obscuring the distinction between idiopathic and secondary NPH. Although other radiographic criteria such as acute callosal angle and disproportionately enlarged subarachnoid space hydrocephalus (DESH) are also supportive of an iNPH diagnosis, their absence is typically not used to exclude patients from further diagnostic evaluation (21). Consequently, the population of patients undergoing evaluation for iNPH and potential shunt placement is highly heterogeneous, which may contribute to the observed variability in treatment outcomes.

A large number of patients are referred for iNPH evaluation after seeking neurological expertise for cognitive decline and gait/balance changes, with findings of ventriculomegaly on subsequent neuroimaging. However, the gait disturbance, cognitive impairment, and urinary dysfunction in iNPH overlap substantially with other neurological disorders such as AD, PD and DLB (22). In the general population, the prevalence of Parkinson's disease is around 3.1% in the 75–84 age range (23), AD is 13.2% between 75 and 84, and DLB is about 5% of all dementia cases in patients above the age of 75 (24). These prevalence rates will also likely shift over time in parallel with advancements in diagnostic testing.

**Figure 1:**
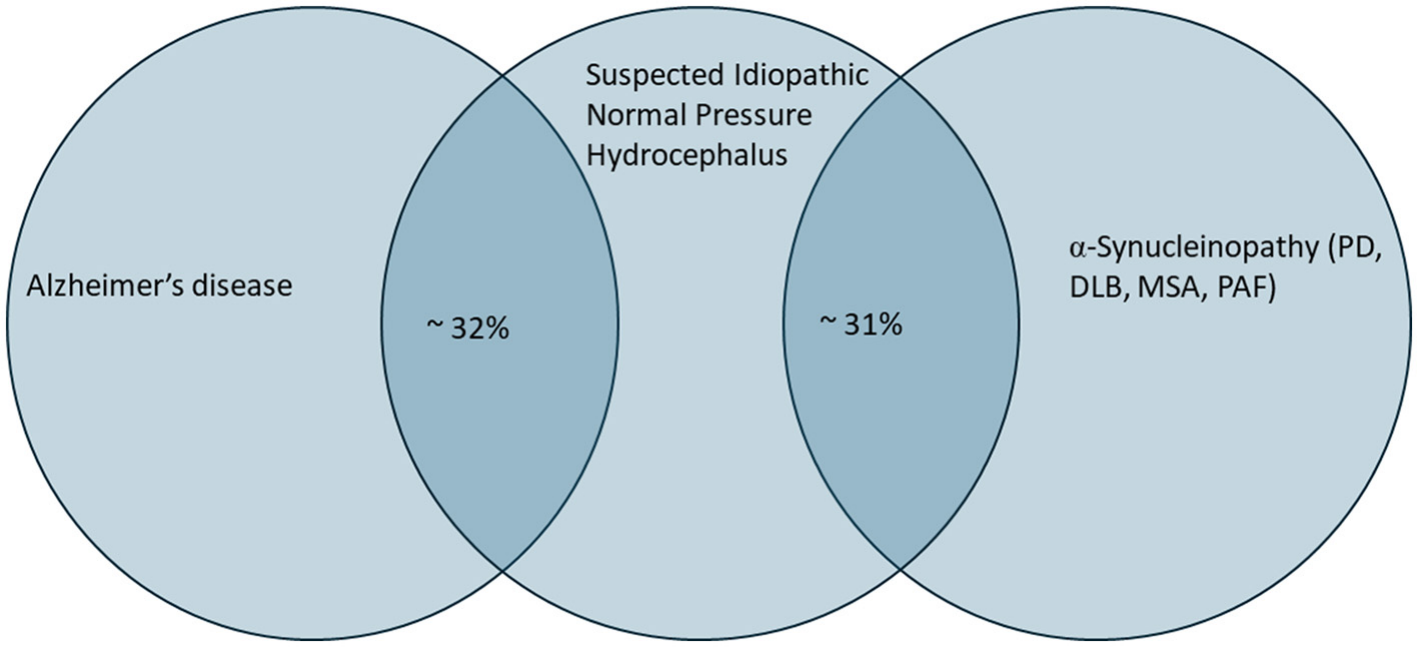
Diagram showing the overlap between patients presenting for evaluation of idiopathic normal pressure hydrocephalus (iNPH) and the percentage of these patients with low Aβ_42/40_ and positive α-synuclein skin biopsy results, indicative of likely Alzheimer's disease and the presence of an α-synucleinopathy, respectively. In our own clinical cohort, we identified 29/89 (32%) with low Aβ_42/40_ ratios and 24/77 (31%) of patients with positive phosphorylated α-synuclein. Of note, although the diagram does not show overlap of all three conditions, 4% (3/72) of patients had positive CSF biomarkers and skin biopsy results.

In a study of 142 iNPH patients treated with shunting, 19% were found to have concurrent histopathological findings consistent for Alzheimer's pathology on intraoperative brain biopsy (25). Across multiple studies, the incidence rates of AD in iNPH are reported to be anywhere from 19 to 68% (26). Using a decreased amyloid-beta (Aβ) 42/40 ratio in CSF as an indication of AD, we have found that approximately 32% (*n* = 29/89) of patients evaluated by high volume lumbar puncture for iNPH had positive CSF biomarkers (Figure 1). Other studies support the high prevalence of positive AD CSF biomarkers in iNPH and an association with worse performance on memory tests (27). Although there is currently no clear evidence of a causal association, it has been suggested that impaired CSF dynamics could compromise the clearance of metabolic waste products and toxic proteins leading to a buildup of Aß deposits (28) whereas increased deposition of Aß in the meninges may increase resistance to CSF outflow (29). Just as likely, patients may have shared risk factors which are common to both disorders, such as cerebrovascular disease or compromised CSF clearance pathways. In support of the latter hypothesis, a Mendelian randomization approach using genome-wide association studies in the European population concluded that there is no evidence of a causal association between the two disorders (26).

Similar to the correlation between iNPH and AD, there is significant overlap in the symptoms of iNPH and α-synucleinopathies, which include PD, DLB, multiple system atrophy (MSA), pure autonomic failure (PAF), and REM sleep behavior disorder (RBD). Utilizing α-synuclein seed amplification assays from CSF (RT-QuIC assays), 20%−33% of patients tested positive for α-synuclein pathology in one study (30, 31) whereas 14% of patients were found positive in an earlier study involving fifty patients (32). The seed assay which involves an amplification step is less sensitive than direct immunofluorescence via skin biopsy (33), which has a range of sensitivities from 93 and 100% for detecting PD, MSA, DLB and PAF (34). In our clinic, we employed the α-synuclein biopsy test during our evaluation of iNPH patients based on clinical suspicion and patient preference. We found that 31% (*n* = 24/77) of patients tested positive, as indicated by at least one positive biopsy (Figure 1). Rarely patients were positive for both CSF AD biomarkers and α-synuclein (*n* = 3/72).

## Clinical features suggesting coexisting Alzheimer's disease or α-synucleinopathy

During the initial evaluation of suspected iNPH, the differential diagnosis should include AD, PD and DLB. iNPH typically presents with gait disturbance before the onset of cognitive decline (35). Accordingly, when cognitive impairment is the presenting or predominant concern and gait has limited impact on quality of life, additional testing to exclude AD or DLB is warranted. Marked short-term memory deficits with relatively preserved executive function and attention further heighten suspicion for AD (36). Incorporating CSF AD biomarkers is readily compatible with the standard iNPH work-up which typically involves a high-volume lumbar puncture (LP) or tap test, external lumbar drainage, or lumbar infusion testing. Of note, CSF Aβ concentrations are often reduced in iNPH, and Aβ42 concentrations alone are found to be consistently lower. Consequently, utilization of the Aβ42/40 ratio, and/or total and phosphorylated Tau can help differentiate AD from iNPH (37).

Parkinsonian features such as bradykinesia and postural instability are frequently found in iNPH patients at a prevalence rate around 70% (38), whereas fewer iNPH patients exhibit rigidity and tremor (especially resting tremor) (39). The cause of parkinsonism in iNPH is not fully understood but is thought to result from potential disruption to the nigrostriatal dopaminergic pathway and downregulation of dopamine receptors in the striatum and putamen (40, 41). Differentiating between iNPH and PD can therefore be challenging (42). The gait pattern in iNPH may share similarities with PD but there are also distinctive features in both conditions. For example, PD usually manifests as a narrow-based gait with reduced arm swing responsive to external cues and sometimes asymmetric, whereas iNPH is broad-based with reduced foot clearance (43). Bulbar symptoms, including dysphagia, dysarthria, sialorrhea and hypomimia, although still present in iNPH to some extent (44), are more indicative of PD. Non-motor manifestations such as apathy, depression and anxiety are common in both, whereas fatigue is often more disabling in PD (45).

In general, careful attention to these clinical subtleties may be quite helpful in raising suspicion for a comorbid neurological disorder in patients with suspected iNPH. Additional atypical symptoms that support α-synuclein testing in patients presenting for iNPH evaluation also include hallucinations, orthostatic hypotension, fluctuating cognition and sleep disturbances.

## Impact of co-existing Alzheimer's disease or α-synucleinopathy on shunt outcomes

As noted, a meaningful subset of patients who improve with provocative testing for iNPH either fail to benefit from shunting or improve only transiently before regressing for unclear reasons (12, 13, 15). It is therefore essential to analyze outcomes among patients with comorbid neurodegenerative disorders in order to determine how their coexistence influences both the magnitude and durability of benefits from shunting. Many studies have examined the effects of comorbid AD on shunt outcomes in iNPH with mixed results. At present, there is no consensus on whether or not patients with iNPH and comorbid AD should pursue or forego shunting. Some studies suggest the clinical response to shunting does not change with the presence of AD pathology (46, 47). Cognitive improvements may be blunted (48), whereas some patients with coexisting AD pathology may be less likely to respond to a tap test (49), and may be less responsive to shunting overall (50). There is also some evidence that shunting can reduce the risk of later developing AD (51). A conservative approach involves a shared discussion between patient and provider acknowledging that coexisting AD may potentially result in a less robust response to shunting. iNPH clinicians should also consider how surgical intervention may influence a patient's eligibility for AD anti-amyloid immunotherapies, since these are contraindicated in any patients with a prior cerebral hemorrhage greater than 1cm in diameter (52) and MRI artifacts due to programmable valves may affect monitoring for amyloid-related imaging abnormalities (ARIA). Although the presence of a shunt is not part of the exclusion criteria, many providers are reluctant to administer these therapies as a result of the increased perception of risk.

**Figure 2:**
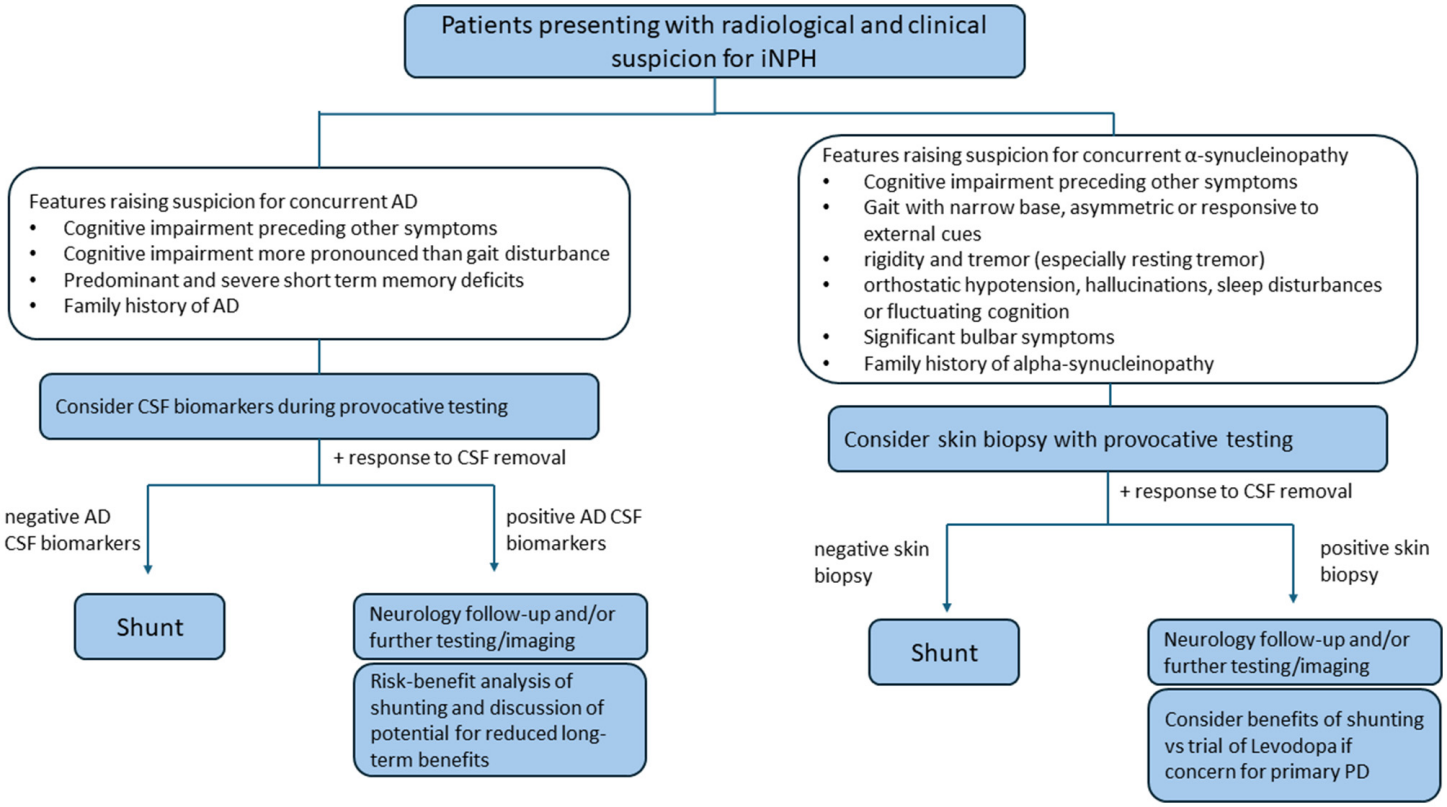
Diagram integrating α-synuclein and Alzheimer's disease cerebrospinal fluid testing in the iNPH evaluation workflow. In the diagram, provocative testing may refer to a high-volume lumbar puncture and/or external lumbar drain.

Unlike in AD, there is less data on shunt outcomes in patients with coexisting α-synucleinopathies. However, using the α-synuclein seed amplification assay from CSF in a cohort of iNPH patients, no difference in shunt surgery outcomes were noted in patients with and without α-synuclein seeding activity (30, 32). Although shunting should therefore still be offered to patients irrespective of α-synuclein status, it may also be helpful to discuss a trial of levodopa if there is suspicion for PD primarily and a less robust response to temporary CSF diversion. A framework for including AD and α-synuclein testing in patients presenting for iNPH evaluation is summarized in Figure 2.

## Discussion

It is estimated that the number of patients diagnosed with iNPH will increase over the next few decades. For example, in Germany, the incidence of iNPH diagnoses increased by 48% between 2005 and 2022 (5). A large part of this increase may be a result of a greater awareness of the condition at both the level of the general public and health care providers (53). It will therefore be important to further refine our knowledge of the interplay between iNPH and other neurological disorders in order to understand how concomitant neurological conditions may affect long term surgical outcomes in this patient population. The specific surgical outcomes expected in this population are also still being refined, as long-awaited results from the Placebo-controlled efficacy in iNPH shunting (PENS) trial become available (4).

This perspective paper underscores the clinical overlap among iNPH, AD and α-synucleinopathies like PD and DLB, drawing on analyses from our iNPH cohort. We advocate incorporating diagnostic testing for common comorbid neurodegenerative disorders into the routine iNPH evaluation, particularly given recent gains in the accuracy and availability of adjunctive assays. While CSF biomarkers for AD are frequently obtained, emerging blood-based tests show strong promise and will likely become standard in neurological practice. Wider adoption of these diagnostics should clarify how concomitant neurodegenerative disease affects shunt outcomes, thereby refining future patient selection and counseling. Figure 2 highlights some of the clinical features that raise suspicion for coexisting pathology and where α-synuclein and CSF biomarker testing may be especially high yield. Given that ≥25% of patients may harbor a concurrent disorder, a case can also be made for offering such testing to all individuals evaluated for iNPH. Notably, patients may test positive on CSF biomarkers or α-synuclein assays even in the absence of suggestive clinical features: for instance, α-synuclein assays can be positive prior to symptom onset (54).

It is important to note that imaging can also provide supportive evidence for AD, PD and LBD without the need for a lumbar puncture or skin biopsy. Fluorodeoxyglucose Positron Emission Tomography (FDG-PET) and amyloid/tau PET are often used in AD, and 123I-ioflupane (DaTscan) SPECT imaging may be used in PD and DLB. Other imaging modalities such as MRI mean diffusivity from diffusion tensor imaging (DTI) have also been shown to differentiate between iNPH and AD, PD and DLB with 86% sensitivity and 96% specificity (55). However, in addition to the use of radioactive tracers, these imaging studies are resource intensive, less accessible and cannot be readily integrated into the standard iNPH outpatient clinical workflow. Alternatively, CSF biomarkers and α-synuclein seed amplification assays and/or skin biopsy testing can be easily incorporated into the normal iNPH evaluation pathway.

In summary, a better understanding of how co-existing neurological disorders influence the response to permanent CSF diversion in iNPH is critical for optimizing both patient selection and outcomes. With recent advances in diagnostic testing for concomitant conditions such as AD and α-synucleinopathies, we propose incorporating these tools into the routine evaluation of iNPH. Although we found that 31% of patients undergoing evaluation for iNPH tested positive for α-synuclein, patients were more likely to want the biopsy when there was existing suspicion for a concomitant α-synucleinopathy or positive family history, which may have biased our sample toward a higher proportion of positives. In addition, our AD analysis focused specifically on the Aβ42/40 ratio. While we present a flow diagram highlighting clinical features that should prompt consideration of alternative neurological diagnoses, these tests may also be offered to all patients, resources permitting, to avoid any bias. Ultimately, multicenter collaborations with larger sample sizes will be essential to generate robust, generalizable data to guide future surgical decision-making in iNPH.

## Data Availability

The raw data supporting the conclusions of this article will be made available by the authors, without undue reservation.
